# Diffuse GFAP Immunopositivity in the Oligodendrocyte-like Component of Pilocytic Astrocytoma Distinguishes It from Mimickers

**DOI:** 10.3390/diagnostics12071632

**Published:** 2022-07-05

**Authors:** Murad Alturkustani

**Affiliations:** Department of Pathology, Faculty of Medicine, King Abdulaziz University, Jeddah 22254, Saudi Arabia; alturkustani.murad@gmail.com; Tel.: +966-50-093-6683

**Keywords:** central neurocytoma, dysembryoplastic neuroepithelial tumors, GFAP, oligodendrocyte-like, oligodendroglioma, pilocytic astrocytoma

## Abstract

Pilocytic astrocytoma with a predominant oligodendrocyte-like component can be difficult to distinguish from oligodendroglioma, dysembryoplastic neuroepithelial tumors (DNTs), central neurocytoma, and ependymoma (clear cell phenotype). The utility of GFAP immunostaining in this context is not well discussed. All cases with a diagnosis of pilocytic astrocytoma were retrieved from the pathological archives along with the following information: age, sex, and pathological description. The GFAP immunostaining was scored as score 1 (<25%), score 2 (25–50%), score 3 (50–75%), and score 4 (>75%). The comparison group included oligodendrogliomas, DNTs, ependymomas, and central neurocytomas. All 26 cases (16 males and 10 females) of pilocytic astrocytoma showed strong and diffuse (score 4) GFAP immunostaining in the neoplastic cells of both the solid fibrillary and oligodendrocyte-like components. The staining pattern in the neoplastic round cells in the oligodendrocyte-like areas was perinuclear cytoplasmic with no processes. In the comparison group, GFAP immunostaining was mostly restricted to the reactive astrocytes in the background. Focal areas of the neoplastic cells showed scores of 1–3 in the neoplastic cells, but the staining pattern was different from those in pilocytic astrocytoma. In the setting of tumors with predominant oligodendrocyte-like areas, the GFAP immunostaining score and pattern help distinguish pilocytic astrocytoma from its mimickers.

## 1. Introduction

Pilocytic astrocytoma is a biphasic neoplasm with compact fibrillary piloid areas containing Rosenthal fibers and loose microcystic areas containing round oligodendrocyte-like cells and eosinophilic granular bodies. Some tumors show a predominant oligodendrocyte-like pattern [1]. These represent a challenge to distinguish from other CNS tumors with oligodendrocyte-like areas such as oligodendroglioma, dysembryoplastic neuroepithelial tumors (DNT), ependymoma (clear cell phenotype), and central neurocytoma.

The distinction between tumors in this category depends on morphological examination, immunostaining characteristics, and molecular alterations. The molecular alterations are more specific for the diagnosis, but their availability is limited to advanced laboratories. Immunostaining is more readily available in most laboratories. In tumors with oligodendrocyte-like cells, the immunopositivity for IDH is diagnostic of oligodendroglioma [2]. Ependymoma (clear cell phenotype) can show EMA and CD99 immunopositivity and scattered oligodendrocyte transcriptional factor-2 (OLIG2) immunopositive cells in the neoplasm [3]. Immunostaining of synaptophysin (a neuronal marker) is one of the essential diagnostic criteria for neurocytoma [4], but it lacks specificity as it is also reported in pilocytic astrocytoma [5], oligodendroglioma [6], and DNET [6]. These limited immunostains may not be enough in complex cases, and other immunostains could help work up this differential diagnosis.

Glial fibrillary acidic protein (GFAP) is diffusely and strongly immunopositive in pilocytic astrocytoma [1,5]. However, its utility in the differential diagnosis of tumors with oligodendrocyte-like areas is not well defined in the literature. Therefore, this study aims to determine the utility of the GFAP immunostaining pattern in supporting the diagnosis of pilocytic astrocytoma in cases with a predominant oligodendrocyte-like component.

## 2. Materials and Methods

The institution’s ethical committee granted the ethical approval to perform this study. I searched in the pathological archives for all cases with a pathological diagnosis of pilocytic astrocytoma, oligodendroglioma, DNT, ependymoma (clear cell phenotype), and central neurocytoma during the 13 years starting from 2008. The following steps were performed: (1) Retrieved the pathological slides with available stains and immunostains. (2) Retrieved the clinical information (age and sex), pathological description, diagnosis, and immunostaining results from the pathological report. The retrieved reports and slides were examined for the following features: diagnosis and the immunostaining characteristics of GFAP and other performed immunostains. These immunostains were available in some cases to confirm or exclude a possible diagnosis and included: anti-EMA, anti-OLIG2, anti-IDH1-R132H, anti-synaptophysin, anti-neurofilament protein, anti-Ki67, and anti-P53 antibodies.

The diagnoses included cases that were confirmed to fulfill the essential diagnostic criteria in the fifth edition of the WHO classification of CNS tumors for each entity. Case 36 was discussed in detail in a previous publication [7].

The inclusion criteria were the presence of an oligodendrocyte-like area in the tumor and the availability of GFAP immunostain to examine these areas. Cases that did not fulfill these criteria were excluded.

The immunostaining procedure was performed according to the laboratory protocol at the time of diagnosis. The samples were fixed in formalin and embedded in paraffin. The laboratory cut the sections at 4 µm thickness and used the BenchMark ULTRA automated stainer from Ventana. The anti-GFAP antibody was clone EP672Y, which is ready to use. The staining procedure for the immunostaining was performed according to the manufacturer’s recommendations for the immunostaining of formalin-fixed, paraffin-embedded tissue. The steps were automated in the machine. However, the most important steps were antigen retrieval by heat, the prediluted primary antibody application, the utilization of the polymer detection kit, and the use of hematoxylin as the counterstain. In addition, the procedure included a quality check for the antibody staining, comparing positive and negative controls. The staining was evaluated using an Olympus BX53 microscope, and the images were taken with the Olympus cellSens entry program using the Olympus SC180 camera.

**Figure 1:**
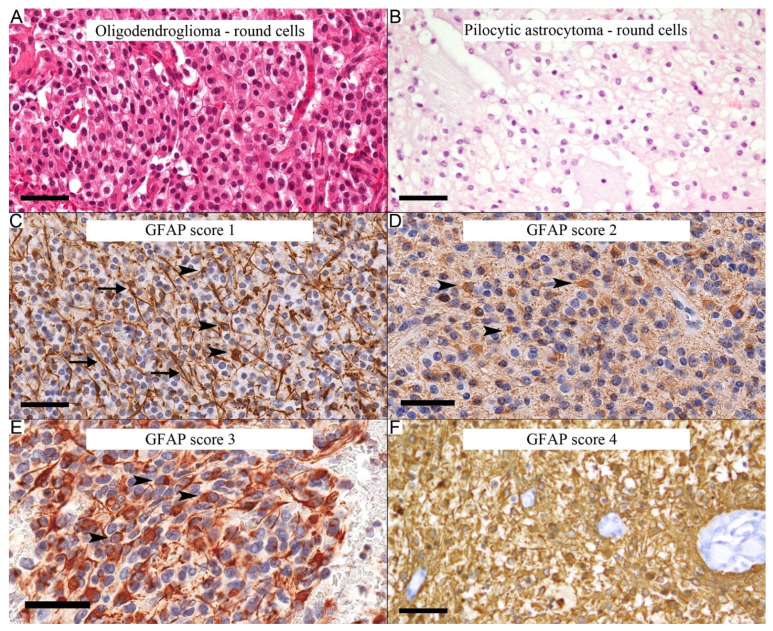
Definition of the oligodendrocyte-like areas and GFAP scores. (**A**) Oligodendroglioma with round neoplastic cells. (**B**) Oligodendrocyte-like area in pilocytic astrocytoma. GFAP scoring system (**C**–**F**). (**C**) Score 1: GFAP-staining cells in less than 25% of neoplastic cells. Most stained cells here are reactive astrocytes (arrows), whereas most neoplastic cells are negative (arrowheads). (**D**) Score 2: GFAP-staining cells in 25–50% of the neoplastic cells. (**E**) Score 3: GFAP staining in 50–75% of the neoplastic cells (arrowheads). (**F**) Score 4: GFAP staining in more than 75% of the neoplastic cells. Scale bars: 50 µm (**A**–**F**).

Oligodendrocyte-like areas are neoplastic cells with monomorphic round nuclei surrounded by perinuclear clearing (Figure 1A,B). The area of interest of the GFAP immunostain was determined by examining the whole slide and selecting the most representative area. This area corresponded to the areas with a prominent oligodendrocyte-like component and the highest GFAP immunostaining. The selected area was then examined under high magnification (40X objective). The GFAP percentage in tumor cells was visually estimated in these areas by comparing neoplastic immunopositive cells to all tumor cells in the field and given a GFAP score. The scoring system was as follows: score 1: staining in less than 25% of the neoplastic cells, score 2: staining in 25% to less than 50% of the neoplastic cells, score 3: staining in 50% to 75% of the neoplastic cells, and score 4: staining in more than 75% of the neoplastic cells. The author was blinded to the diagnosis when performing the scoring. However, the histological features on the evaluated slides could have revealed the diagnosis during the scoring.

The hypothesis for this study was that the GFAP immunostaining score of oligodendrocyte-like areas in pilocytic astrocytoma is different from other CNS tumors in the differential diagnoses (comparison group). In this regard, inferential statistic (chi-square) was used, and the results were interpreted at the 0.05 level of statistical significance.

## 3. Results

There were 48 cases diagnosed with pilocytic astrocytoma during this period. Only 26 cases fulfilled the inclusion criteria. There were 16 males and 10 females. The age of patients in this cohort ranged from 17 months to 31 years, with a mean of 11.25 years. The comparison group included five cases of oligodendroglioma, three cases of central neurocytoma, three cases of supratentorial ependymoma (clear cell phenotype), and two cases of DNT. All oligodendroglioma cases were immunopositive for IDH1-R132H immunostains, the three cases of central neurocytoma were immunopositive for synaptophysin, and the three cases of ependymoma showed at least focal dot-like cytoplasmic immunopositivity for EMA. As molecular tests were not performed on these cases, the designation of “not otherwise specified (NOS)” was added to the diagnosis of oligodendroglioma and supratentorial ependymoma as recommended by the WHO classification. The clinical information for these cases and the GFAP staining score are shown in Table 1.

**Figure 2:**
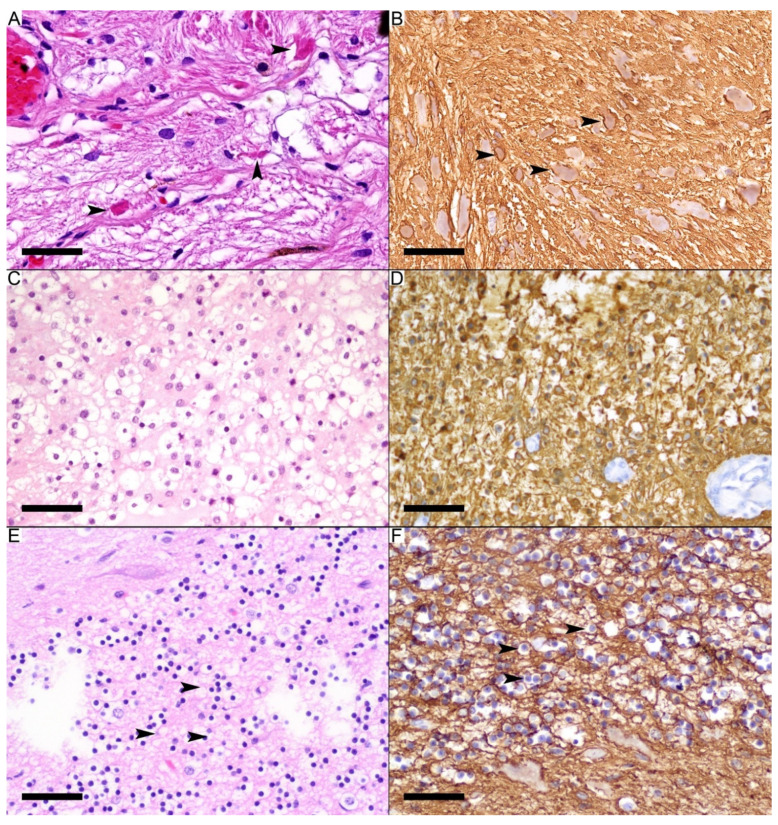
GFAP staining in pilocytic astrocytoma. (**A**) Solid fibrillary area in pilocytic astrocytoma with Rosenthal fibers (arrowheads). (**B**) Diffuse and strong GFAP immunostaining in the elongated neoplastic cells. The Rosenthal fibers are within the astrocytic processes (arrowheads). (**C**) Microcystic area with oligodendrocyte-like neoplastic cells. (**D**) Diffuse and strong GFAP immunostaining (score 4) in the round cells. The immunostaining is mainly a cytoplasmic rim around the nucleus with no processes. (**E**) The granular cell layer of the cerebellum appears as round blue cells with perinuclear clearing (arrowheads). (**F**) GFAP staining in the granular cell layer highlights background staining, whereas the round cells are immunonegative for the GFAP immunostain (arrowheads). Scale bars: 50 µm (**A**–**F**).

GFAP immunostaining was strongly and diffusely positive in both the solid fibrillary component and the oligodendrocyte-like component in all pilocytic astrocytoma cases. The solid fibrillary areas contained elongated bipolar cells, strongly immunopositive for GFAP (Figure 2A). Rosenthal fibers in these areas were surrounded by GFAP immunostaining (Figure 2B), confirming the intracellular location of these fibers. The round neoplastic cells (oligodendrocyte-like) showed diffuse perinuclear cytoplasmic staining with no processes (Figure 2C,D), differentiating them from occasional reactive astrocytes with cytoplasmic processes in the background. The morphology of five cases (cases 21–26) of pilocytic astrocytoma was predominantly of the oligodendrocyte-like pattern. Other round cells in these specimens may show morphological overlap with the neoplastic cells, but they did not express GFAP immunostaining. These include oligodendrocytes with perinuclear clearing, immunonegative for GFAP, and the granular cell layer of the cerebellum, where GFAP immunostaining was restricted to the background glial cells (Figure 2E,F).

A two-tailed chi-square test signified that the GFAP score for pilocytic astrocytoma significantly differs from the GFAP scores for other neoplasms (X2 = 34.63, df = 1, *p* = 0.0001). In addition, the findings indicated the sensitivity of the GFAP score for pilocytic astrocytoma. The mean and range for GFAP scores were 4 and 0, respectively. However, the mean for other neoplasms ranged between 1 and 3. This finding indicated that the specificity of the GFAP score for pilocytic astrocytoma was more than for other neoplasms.

**Figure 3:**
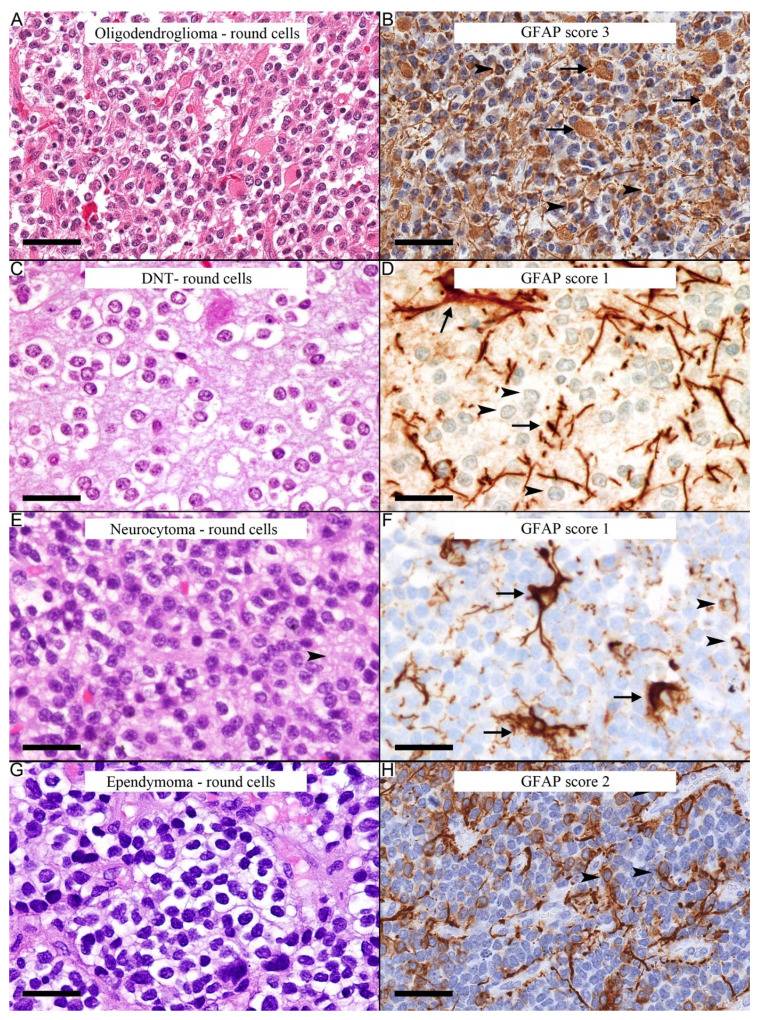
GFAP staining in oligodendrocyte-like areas in other tumors. (**A**) Oligodendroglioma with an area rich in neoplastic gliofibrillary oligodendrocytes and minigemistocytes. (**B**) GFAP highlights the gliofibrillary oligodendrocytes (arrowheads) and minigemistocytes (arrows). (**C**) Round neoplastic cells with perinuclear clearing in DNT. (**D**) GFAP is mainly in the background’s reactive astrocytes (arrows), whereas the round neoplastic cells are immunonegative (arrowheads). (**E**) Central neurocytoma with round neoplastic cells with neuropil in the background (arrowhead). (**F**) GFAP immunostain is immunopositive in scattered neoplastic cells (arrowheads) and the reactive astrocytes in the background (arrows). (**G**) Round neoplastic cells in ependymoma (clear cell phenotype). (**H**) GFAP highlights neoplastic cells with perivascular accentuation for GFAP (arrowheads). Scale bars: 50 µm (**A**–**H**).

GFAP in the comparison group was mainly in the reactive astrocytes in the background. The GFAP positive immunostaining in neoplastic oligodendrocyte-like cells, when present, was less than score 4, and the pattern of staining was different. In oligodendrogliomas, in three out of five cases, the GFAP staining was mainly in the background reactive astrocytes. Focal areas in two out of five cases contained predominant gliofibrillary oligodendrocytes and minigemistocytes, strongly positive for GFAP immunostaining. The GFAP staining pattern of gliofibrillary oligodendrocytes was a perinuclear cytoplasmic rim, similar to the staining pattern in the oligodendrocyte-like cells in pilocytic astrocytoma, but pilocytic astrocytoma cells may show a cytoplasmic process. Minigemistocytes showed cytoplasmic round inclusion-like GFAP immunoreactivity (Figure 3A,B).

GFAP showed minimal to occasional immunostaining of the round neoplastic cells in DNTs (Figure 3C,D) and neurocytomas (Figure 3E,F). The staining in the ependymoma (clear cell phenotype) was variable and showed concentration in the perivascular areas (Figure 3G,H). The characteristic perinuclear cytoplasmic rim-like staining observed in the oligodendrocyte-like areas in pilocytic astrocytoma can occasionally be present as rare or few neoplastic cells among the neoplastic cells of these tumors.

## 4. Discussion

Pilocytic astrocytoma with predominant oligodendrocyte-like areas can pose a challenge in the diagnosis. The differential diagnoses for this pattern include oligodendroglioma, DNT, central neurocytoma, and ependymoma (clear cell phenotype). Some immunostains can be helpful in this setting as immunopositivity for IDH is diagnostic of oligodendroglioma [2], and EMA cytoplasmic dot-like and ring staining are consistent with ependymoma [3]. OILG2 is diffusely positive in this list of differential diagnoses except for ependymoma, which shows no immunopositivity or only scattered immunopositivity in the neoplastic cells [3]. GFAP is known to be diffusely and strongly immunopositive in pilocytic astrocytoma [1]. However, its utility in the differential diagnosis of tumors with oligodendrocyte-like areas was not well defined in the literature.

In this work, diffuse and strong GFAP immunostaining in more than 75% (score 4) of neoplastic cells was consistent in all cases of pilocytic astrocytoma. Furthermore, the staining pattern in oligodendrocyte-like neoplastic cells is also different from that of oligodendrocyte-like neoplastic cells in other tumors.

Morphological assessment is usually sufficient for diagnosing pilocytic astrocytoma when the characteristic biphasic pattern is present. However, pilocytic astrocytoma with a predominant oligodendrocyte-like pattern may need additional immunostaining to differentiate it from its mimickers. The distinction between the entities in the differential diagnosis is crucial as they have different CNS WHO grades, treatments, and prognoses. The results of molecular studies are more specific but not readily available in most laboratories worldwide.

Common immunostains that can be used in approaching CNS tumors with an oligodendrocyte-like pattern include IDH, neuronal markers (synaptophysin, neurofilament protein, and NeuN), EMA, and GFAP. GFAP is a general marker of glial differentiation, and the staining pattern was not thoroughly investigated to distinguish between the tumors in the differential diagnoses. GFAP is a type III intermediate filament. In the developing brain, it stains radial glia [8], immature oligodendrocytes [9,10], and two subtypes of fetal ependymal cells [11]. In the normal postnatal brain, GFAP stains protoplasmic astrocytes, fibrous astrocytes, Bergmann glia, subependymal astrocytes, pituicytes [8], and neural stem cells [12] but not oligodendrocytes or mature ependymal cells [11].

In clinical practice, GFAP is commonly used as a classical marker of astrocytoma to confirm glial differentiation [12]. It is diffusely expressed in pilocytic astrocytomas [5,12] and higher-grade astrocytomas [12]. As GFAP is expressed in different cells and grades of astrocytoma, it is considered a non-specific marker of glial differentiation [5]. This work proposes a novel specific utility interpreting GFAP staining in CNS tumors with predominant oligodendrocyte-like areas. Score 4 GFAP (more than 75%) staining in neoplastic cells in these areas is consistent with a diagnosis of pilocytic astrocytoma, whereas a lower GFAP score is not specific and does not distinguish between the tumors in the differential diagnoses.

IDH mutation and 1p/19q codeletion are diagnostic of oligodendroglioma, but “pediatric-type” oligodendroglioma lacks these alterations, representing a challenge for differential diagnosis in this setting. Normal and neoplastic oligodendrocytes typically are immunonegative for GFAP. The reactive GFAP-expressing cells in oligodendroglioma are limited to the reactive astrocytes in the background and two forms of transitional neoplastic oligodendrocytes (gliofibrillary astrocytes and minigemistocytes) [2,9]. The staining patterns in these cells are cytoplasmic with some processes, and small cytoplasmic round inclusion-like, respectively.

The presence of the specific glioneuronal element is diagnostic of DNT [13]. These are formed by floating neurons and oligodendrocyte-like cells, characteristically immunopositive for OLIG2 whereas it was immunonegative for GFAP [13,14]. The round neoplastic cells in central neurocytoma show immunostaining evidence of neuronal differentiation, most commonly by the diffuse synaptophysin immunostaining. GFAP immunostaining in central neurocytoma is variable but typically restricted to the background reactive astrocytes [4]. Synaptophysin staining is not specific and can be seen in pilocytic astrocytoma [5], DNT, and oligodendroglioma [6].

Dot-like and ring cytoplasmic staining for EMA is characteristic of ependymoma but is not present in all cases [3]. GFAP staining in ependymoma is common but usually not diffusely positive in all neoplastic cells, and it shows perivascular accentuation [3,15].

The diffuse and strong GFAP immunostaining in the round oligodendrocyte-like neoplastic cells in pilocytic astrocytoma was in the form of a thin rim of perinuclear cytoplasmic staining. These cells were the main component in the round neoplastic cells in pilocytic astrocytoma, and few similar cells can be present in other tumors in the differential diagnoses. The presence of these cells in other tumors can be explained by tumor heterogeneity, where these cells could represent a minor component of the neoplastic cells in these tumors.

Tihan et al. found GFAP is consistently immunopositive in 94/94 pilocytic astrocytoma in which 90/94 showed more than 50% staining in the neoplastic cells (category 3 and 4), 2/94 showed immunostaining in 25–50% of the neoplastic cells (category 2), and the remaining 2 cases showed immunostaining in less than 25% of the neoplastic cells (category 1) [5]. The result in this study is consistent with their observations, as all pilocytic astrocytomas had a GFAP score of 4 (which corresponds to category 4). The lack of pilocytic astrocytomas with a lower GFAP score in this study could be explained by the lower number of cases and the selective evaluation of oligodendrocyte-like components.

When assessing GFAP staining in these samples, the pathologist should be cautious to differentiate between the neoplastic and non-neoplastic oligodendrocyte-like cells. As demonstrated in this study, non-neoplastic oligodendrocytes and granular cells can show round nuclei with perinuclear clearing, and they are consistently immunonegative for GFAP.

The limitation of this study is related to the small number of examined cases, the potential selection bias of the comparison group, and the portions of the tumor reviewed. The number of cases in the comparison group is low in this study, but the results of GFAP immunostaining are compatible with what is known in the literature. As this study has a limited number of pilocytic astrocytoma cases, the possibility of pilocytic astrocytomas with a lower GFAP score cannot be excluded. As the available clinical information does not include specific information about the patients’ genetic alterations, the difference in GFAP staining in NF1-associated versus non-NF1-associated pilocytic astrocytomas cannot be assessed.

## 5. Conclusions

Strong and diffuse GFAP immunostaining in most neoplastic cells is a consistent finding in pilocytic astrocytoma. In the setting of CNS tumors with oligodendrocyte-like areas, diffuse and strong cytoplasmic GFAP immunostaining in these cells favor the diagnosis of pilocytic astrocytoma. Further studies, including more cases of the differential diagnoses for CNS tumors with an oligodendrocyte-like pattern, are required to confirm the utility of GFAP immunostaining in this setting.

## Figures and Tables

**Table 1:** Clinical data and GFAP staining score.

No.	Age in Years	Sex	Location	Diagnosis	GFAP Score
1	4	F	Posterior fossa	Pilocytic astrocytoma	4
2	27	M	Cerebellum	Pilocytic astrocytoma	4
3	11	F	Cerebellum	Pilocytic astrocytoma	4
4	4	F	Cerebellum	Pilocytic astrocytoma	4
5	19	F	Left parietal	Pilocytic astrocytoma	4
6	29	F	Cerebellum	Pilocytic astrocytoma	4
7	7	F	Left parietal	Pilocytic astrocytoma	4
8	19	M	Unknown	Pilocytic astrocytoma	4
9	18	M	Cerebellum	Pilocytic astrocytoma	4
10	5	M	Right parietal	Pilocytic astrocytoma	4
11	4	F	Cerebellum	Pilocytic astrocytoma	4
12	2	M	Cerebellum	Pilocytic astrocytoma	4
13	13	M	Right parietal	Pilocytic astrocytoma	4
14	4	M	Cerebellum	Pilocytic astrocytoma	4
15	31	M	Cerebellum	Pilocytic astrocytoma	4
16	6	M	Cerebellum	Pilocytic astrocytoma	4
17	8	M	Spinal (T10-L1)	Pilocytic astrocytoma	4
18	10	F	Cerebellum	Pilocytic astrocytoma	4
19	23	M	Suprasellar	Pilocytic astrocytoma	4
20	7	F	Cerebellum	Pilocytic astrocytoma	4
21	5	M	Brain stem	Pilocytic astrocytoma	4
22	15	M	Cerebellum	Pilocytic astrocytoma	4
23	3	M	Cerebellum	Pilocytic astrocytoma	4
24	5	M	Right frontal	Pilocytic astrocytoma	4
25	12	F	Cerebellum	Pilocytic astrocytoma	4
26	1.5	M	Cerebellum	Pilocytic astrocytoma	4
27	55	F	Left frontal	Oligodendroglioma, NOS	1
28	49	M	Right frontal	Oligodendroglioma, NOS	2
29	32	M	Right temporal	Oligodendroglioma, NOS	1
30	63	M	Left frontal	Oligodendroglioma, NOS	3
31	38	F	Left frontal	Oligodendroglioma, NOS	2
32	21	M	Intraventricular	Central neurocytoma	1
33	47	F	Intraventricular	Central neurocytoma	1
34	30	F	Intraventricular	Central neurocytoma	1
35	16	F	Third ventricle	Supratentorial ependymoma, NOS	2
36	19	M	Right frontal	Supratentorial ependymoma, NOS	3
37	7	M	Right frontal	Supratentorial ependymoma, NOS	2
38	6	M	Right parieto-occipital	DNT	1
39	14	M	Unknown	DNT	2

DNT: dysembryoplastic neuroepithelial tumors; F: female; M: male; NOS: not otherwise specified.

## Data Availability

All relevant data files are available from the corresponding author (MA) upon request.
